# Supplementary material for: Herring Milt and Herring Milt Protein Hydrolysate Are Equally Effective in Improving Insulin Sensitivity and Pancreatic Beta-Cell Function in Diet-Induced Obese- and Insulin-Resistant Mice
Source: Mar Drugs. 2020 Dec 11;18(12):635. doi: 10.3390/md18120635 (PMC7763884; doi:10.3390/md18120635)
Supplement: Supplementary file 1 [file marinedrugs-18-00635-s001.pdf]

Table S1. Amino acid composition of casein, herring milt dry powder (HMDP) and herring milt protein hydrolysate (HMPH)

AA (mg/g protein)*	Casein	HMDP	HMPH
Asparagine + aspartic acid	69.04	71.43	79.49
Glutamine + glutamic acid	219.89	111.95	102.56
Serine	59.36	46.60	30.77
Glycine	17.88	73.67	89.74
Histidine	23.11	18.42	15.38
Arginine	37.08	324.15	292.31
Taurine	0.00	33.15	30.77
Threonine	41.62	37.32	33.33
Alanine	29.05	44.20	41.03
Proline	120.94	44.04	43.59
Tyrosine	53.92	22.74	25.64
Valine	63.63	42.76	43.59
Methionine	26.51	15.86	17.95
Isoleucine	45.57	23.06	33.33
Leucine	85.76	38.60	43.59
Phenylalanine	40.36	18.26	28.21
Lysine	48.19	17.14	35.90
Cysteine	2.54	8.33	10.26
Tryptophan	15.26	0.48	2.56

∗ Protein was calculates as the sum of all amino acids.

Table S2. Diet composition and calorie information

Ingredient[#]	LFC[&]	HFC	HMDP70	HMPH70
Diet composition				
Casein (80 Mesh)	200	200	60	60
L-Cystine	3	3	3	3
Corn Starch	550	0	0	0
Maltodextrin 10	150	125	125	125
Sucrose	0	68.8	68.8	68.8
Herring Milt Hydrolysate	0	0	0	197.7
Herring Milt	0	0	202.9	0
Cellulose, BW200	50	50	50	50
Lard (96%)	20	245	222.3	223.6
Soybean Oil	25	25	25	25
Mineral Mix S10026	10	10	10	10
DiCalcium Phosphate	13	13	13	13
Calcium Carbonate	5.5	5.5	5.5	5.5
Potassium Citrate, 1 H_2O	16.5	16.5	16.5	16.5
Vitamin Mix V10001	10	10	10	10
Choline Bitartrate	2	2	2	2
Total	1055	774	814	810
Calorie information				
Protein (% kcal)	20	20	20	20
Fat (% kcal)	10	60	60	60
Carbohydrate (% kcal)	70	20	20	20
Energy density (kcal/g)	3.85	5.24	4.91	4.98

[#] Diet formulae of LFC and HFC and the ingredients were obtained from the Research Diets, New Brunswick, NJ, USA.

[&] LFC, low-fat control diet; HFC, high-fat control diet; HMDP70, HFC with 70% casein replaced with the same amount of protein derived from herring milt dry powder; HMPH70, HFC with 70% casein replaced with the same amount of protein derived from herring milt protein hydrolysate.
